# Ultra-High-Resolution Photon-Counting Detector CT Benefits Visualization of Abdominal Arteries: A Comparison to Standard-Reconstruction

**DOI:** 10.1007/s10278-024-01232-5

**Published:** 2024-10-25

**Authors:** Huan Zhang, Yue Xing, Lingyun Wang, Yangfan Hu, Zhihan Xu, Haoda Chen, Junjie Lu, Jiarui Yang, Bei Ding, Weiguo Hu, Jingyu Zhong

**Affiliations:** 1 Department of Radiology, Ruijin Hospital, Shanghai Jiao Tong University School of Medicine, Shanghai, 200025 China; 2 Department of Imaging, Tongren Hospital, Shanghai Jiao Tong University School of Medicine, Shanghai, 200336 China; 3 Siemens Healthineers, Shanghai, 201318 China; 4 Department of General Surgery, Pancreatic Disease Center, Ruijin Hospital, Shanghai Jiao Tong University School of Medicine, Shanghai, 200025 China; 5 Department of Epidemiology and Population Health, Stanford University School of Medicine, Stanford, CA 94305 USA; 6 Department of Biomedical Engineering, Boston University, Boston, MA 02215 USA; 7 Department of Geriatrics and Surgery, Ruijin Hospital, Shanghai Jiao Tong University School of Medicine, Shanghai, 200025 China; 8 Medical Center On Aging of Ruijin Hospital, Shanghai Jiao Tong University School of Medicine, Shanghai, 200025 China

**Keywords:** Computed tomography angiography, Contrast media, Image reconstruction, Image enhancement, Radiation dosage

## Abstract

This study aimed to investigate the potential benefit of ultra-high-resolution (UHR) photon-counting detector CT (PCD-CT) angiography in visualization of abdominal arteries in comparison to standard-reconstruction (SR) images of virtual monoenergetic images (VMI) at low kiloelectron volt (keV). We prospectively included 47 and 47 participants to undergo contrast-enhanced abdominal CT scans within UHR mode on a PCD-CT system using full-dose (FD) and low-dose (LD) protocols, respectively. The data were reconstructed into six series of images: FD_UHR_Br48, FD_UHR_Bv56, FD_UHR_Bv60, FD_SR_Bv40, LD_UHR_Bv48, and LD_SR_Bv40. The UHR reconstructions were performed with three kernels (Bv48, Bv56, and Bv60) within 0.2 mm. The SR were virtual monoenergetic imaging reconstruction with Bv40 kernel at 40-keV within 1 mm. Each series of axial images were reconstructed into coronal and volume-rendered images. The signal-to-noise ratio (SNR) and contrast-to-noise ratio (CNR) of seven arteries were measured. Three radiologists assessed the image quality, and visibility of nine arteries on all the images. SNR and CNR values of SR images were significantly higher than those of UHR images (*P* < 0.001). The SR images have higher ratings in image noise (*P* < 0.001), but the FD_UHR_Bv56 and FD_UHR_Bv60 images has higher rating in vessel sharpness (*P* < 0.001). The overall quality was not significantly different among FD_VMI_40keV, LD_VMI_40keV, FD_UHR_Bv48, and LD_UHR_Bv48 images (*P* > 0.05) but higher than those of FD_UHR_Bv56 and FD_UHR_Bv60 images (*P* < 0.001). There is no significant difference of nine abdominal arteries among six series of images of axial, coronal and volume-rendered images (*P* > 0.05). To conclude, 1-mm SR image of VMI at 40-keV is superior to 0.2-mm UHR regardless of which kernel is used to visualize abdominal arteries, while 0.2-mm UHR image using a relatively smooth kernel may allow similar image quality and artery visibility when thinner slice image is warranted.

**Supplementary Information:**

The online version contains supplementary material available at 10.1007/s10278-024-01232-5.

## Introduction

The CT angiography (CTA) of abdominal arteries serves as the reference standard imaging modality for the diagnosis and risk evaluation of abdominal vascular diseases as well as follow-up after endovascular intervention and surgery [1, 2]. The CTA of abdominal arteries is also necessary for operation planning of abdominal region [3–5]. However, it is still a challenge for CTA examinations to present small segments of abdominal arteries which is important for clinical practice [6, 7]. The variations of the small segments of hepatic and gastroduodenal arteries require different surgical technique and should be visualized before operation to guide the decision-making [8, 9]. The insufficient visualization can lead to unexpected damage to the abdominal arteries and undesired bleeding during operation or even complications such as ischemia and necrosis of the whole organs [10].

Compared to the CTA using conventional CT scanners, the dual-energy CT scanners allows reconstruction of virtual monoenergetic images (VMI) at low kiloelectron volt (keV) to better visualize the abdominal arteries and has been considered as the optimal imaging modality [11, 12]. Furthermore, photon-counting detector CT (PCD-CT) yields direct conversion of incoming photons into electronic signals proportional to their deposited energy, in order to count each individual x-ray photon in the scan. Since the electronic noise would no longer affect the photon count rate, the images quality is still guaranteed with an additional radiation reduction [13–15]. The CTA of abdominal arterials can be realized with lower radiation doses and less iodinated contrast media [16–19]. In addition to the low-keV VMI, the detector architecture of PCD-CT allowed and introduced the ultra-high-resolution (UHR) mode [20, 21]. The UHR images are expected to provide better image quality and higher sharpness for small anatomical structures compared to standard-resolution (SR) images. The potential advantages of UHR in coronary CTA includes reduced blooming artifacts, improve in-stent lumen visibility and stenosis quantification, and better plaque characterization [22–27]. The better image quality of UHR mode was also demonstrated in CTA of femoral arteries and has potential in increasing diagnostic accuracy [28]. Nevertheless, it is unclear whether and how the UHR mode can benefit the visualization of small segments of abdominal arteries.

Therefore, our study aims to investigate the potential benefit of PCD-CT angiography using UHR images in visualization of abdominal arteries in comparison to SR images of low-keV VMI.

## Method

### Study Design and Participants

**Fig. 1:**
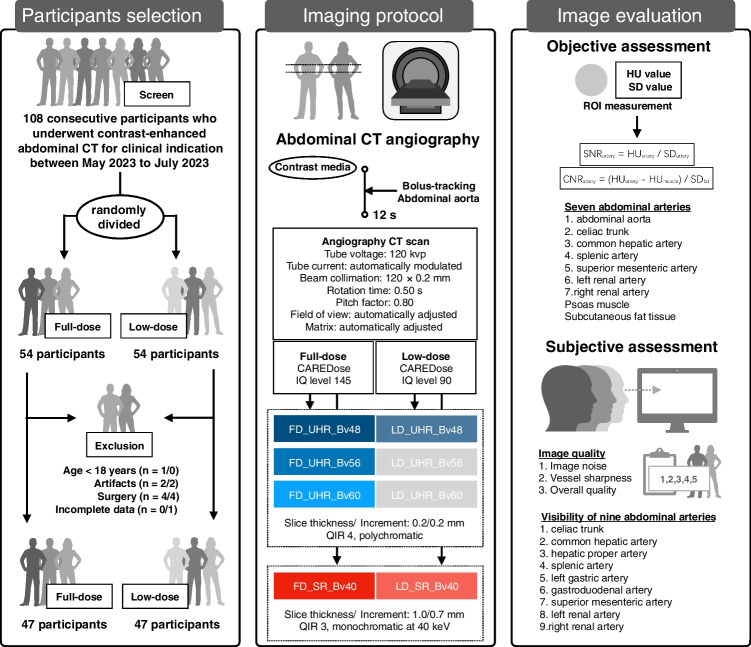
Workflow of the current study

This prospective study has been approved by the institutional ethic review board. The written inform consent were obtained from all participants. The workflow of this study is presented in Fig. 1. We prospectively screened consecutive participants who scheduled to undergo contrast-enhanced abdominal CT for clinical indication between May and July 2023. The participants who fulfilled the inclusion criteria were randomly divided into FD and LD groups, respectively. After the scan, patients in FD and LD group were matched according to gender, age, and body mass index. The following factors were considered acceptable: a deviation of ± 2 years for age and ± 2 kg/m^2^ for body mass index [29, 30]. The exclusion criteria were (a) pregnant, (b) age < 18 years, (c) refuse to consent, (d) allergic history contraindication to contrast media, (e) history of abdominal surgery, (f)  severe motion artifacts, and (g) incomplete data.

### CT Scan and Image Reconstruction

All acquisitions were performed on a PCD-CT system (NAEOTOM Alpha, Syngo.CT version VA50-SP1, Siemens Healthineers). The parameters for UHR scans were tube voltage of 120 kVp, collimation of 120 × 0.2 mm, rotation time of 0.50 s, pitch of 0.8, and within automatic tube current modulation (CARE Dose 4D, Siemens Healthineers) at image quality level of 145 and 90 for FD and LD groups, respectively. The nonionic contrast material (1.5 mL/kg body weight, Ultravist 370, Schering) was administrated using a power injector at a rate of 3.0 mL/s using a pump injector. The arterial phase scan was initiated with a 12-s delay when the abdominal aorta enhancement appeared.

All the data was anonymously retrieved and reconstructed into six series of images: FD_UHR_Bv48, FD_UHR_Bv56, FD_UHR_Bv60, FD_ SR_Bv40, LD_UHR_Bv48, and LD_SR_Bv40 (Table 1). The LD_UHR_Bv56 and LD_UHR_Bv60 images were not reconstructed since our preliminary study indicated that they were not acceptable for clinical use (Supplementary Table S1). The UHR reconstructions were polychromatic images at slice thickness/increment of 0.2/0.2 mm using quantum iterative reconstruction (QIR) at strength level 4 out of 4, within three kernels of Bv48, Bv56, and Bv60. The SR reconstructions were virtual monoenergetic images of kiloelectron volt level of 40-keV at slice thickness/increment of 1.0/0.7 mm, using QIR at strength level 3 out of 4, within a kernel of Bv40. The Bv-type kernels are recommended by the vendor for image reconstruction of body vessel. The larger the value, the sharper the kernel, i.e., the image reconstruction would emphasize more the image sharpness at the expense of image noise. The kernel for SR reconstruction was determined according to the recommendation of the vendor. The kernels for UHR reconstruction were selected to test the potential of UHR for visualization of abdominal arteries. The QIR is a reconstruction algorithm that specifically tailored to PCD-CT and has four strength levels. The strength levels of the QIR in our study were decided according to the recommendation of the vendor and a former research [31]. The field of view and matrix are automatically adjusted for optimal image quality. Each series of axial images were further reconstructed into coronal images and three-dimensional volume-rendered (VR) images using a manufacturer-specific spectral workstation (Syngo.Via, version VB60, Siemens Healthineers).

**Table 1:** CT technique of six series of images

Parameter	FD_UHR_Bv48	FD_UHR_Bv56	FD_UHR_Bv60	FD_SR_Bv40	LD_UHR_Bv48	LD_SR_Bv40
Acquisition						
Tube voltage, kVp	120	120	120	120	120	120
Collimation, mm	120 × 0.2	120 × 0.2	120 × 0.2	120 × 0.2	120 × 0.2	120 × 0.2
Rotation time, s	0.50	0.50	0.50	0.50	0.50	0.50
Pitch	0.80	0.80	0.80	0.80	0.80	0.80
Automatic tube current with CARE Dose 4D at image quality level	145	145	145	145	90	90
Reconstruction						
Field of view and matrix	Automatically adjusted	Automatically adjusted	Automatically adjusted	Automatically adjusted	Automatically adjusted	Automatically adjusted
Thickness, mm	0.2	0.2	0.2	1.0	0.2	1.0
Increment, mm	0.2	0.2	0.2	0.7	0.2	0.7
Reconstruction algorithm	QIR 4/4	QIR 4/4	QIR 4/4	QIR 3/4	QIR 4/4	QIR 3/4
Reconstruction kernel	Bv48	Bv56	Bv60	Bv40	Bv48	Bv40
Energy level, keV	Polychromatic	Polychromatic	Polychromatic	40	Polychromatic	40

*FD*, full dose; *keV*, kiloelectron volt; *LD*, low dose; *QIR*, quantum iterative reconstruction; *SR*, standard-reconstruction; *UHR*, ultra-high-resolution

### Radiation Dose Estimates

The effective tube current, volume CT dose index (CTDIvol, mGy) were automatically recorded and extracted directly from the scanner. The dose-length product (DLP, mGy·cm) was calculated as DLP = CTDIvol × scan length. The effective dose (ED, mSv) was calculated as ED = DLP × conversion coefficient *k*, as the *k* for adult abdominal scans is 0.015 [32]. The size-specific dose estimate (SSDE, mGy) was calculated according to the recommendation [33].

### Objective Image Assessment

The objective image quality assessment was performed by a radiologist with 5 years of experience using the workstation (Syngo.Via, version VB60, Siemens Healthineers) with default tools (Supplementary Note S1). The regions of interest (ROIs) were put on seven arteries (abdominal aorta, celiac trunk, common hepatic artery, splenic artery, superior mesenteric artery, left renal artery, and right renal artery), psoas muscle, and subcutaneous fat tissue, for CT number values and corresponding standard deviation (SD) values [34, 35]. The circular ROIs with a diameter of 3 to 15 mm were put on axial slices that present the arteries, covering the vascular lumen as much as possible while avoiding to touch vascular walls, calcification, thrombus, or artifacts. Then, the ROIs were copied and pasted to other series of images. The SD values of homogeneous anterior abdominal subcutaneous fat tissues at the third lumbar vertebra level was defined as the background noise. The CT number values of psoas muscle were recorded. Each HU and SD value was calculated by averaging the measurements of three consecutive axial image slices. The signal-to-noise ratio (SNR) was calculated as SNR_artery_ = HU_artery_/SD_artery_. The contrast-to-noise ratio (CNR) was calculated as CNR_artery_ = (HU_artery_—HU_muscle_)/SD_fat_ [36].

### Subjective Image Assessment

The subjective image quality assessment was performed by three radiologists with 5, 5, and 6 years of experience, respectively, in the reading room using medical monitors with daily settings (Supplementary Note S2). The image quality was assessed in terms of image noise, vessel sharpness, and overall quality. A 5-point Likert scale was used: 1, unacceptable; 2, suboptimal; 3, acceptable; 4, good; and 5, excellent [37–40]. The visibility of nine arteries (celiac trunk, common hepatic artery, hepatic proper artery, splenic artery, left gastric artery, gastroduodenal artery, superior mesenteric artery, left renal artery, and right renal artery) were evaluated using axial, coronal, volume rendered images. A 5-point Likert scale was defined as follows: 1, no vascular segment was clearly visualized; 2, between 1 and 3; 3, nearly half of the vascular segments were clearly visualized; 4, between 3 and 5; 5, all vascular segments from the trunk to the subsegmental peripheral artery were clearly visualized [35]. The radiologists independently viewed the images blinded to the reconstruction parameters, presenting with a default window width of 700 HU and window level of 80 HU. The window width, window level, viewing distance, and viewing time were not restricted.

### Statistical Analysis

The statistical analysis was carried out using R language version 4.1.3 with RStudio version 1.4.1106 by a radiologist with 6 years of experience in radiological research. The continuous and categorical variables were described as mean ± SD (interquartile range or range), and frequency (percentage), respectively. The one-way analysis of variance and Kruskal–Wallis *H* test were used to compare objective measures and subjective ratings of six series of images, respectively. The post hoc pairwise comparisons were conducted if a significant difference was found. A two-tailed alpha level of 0.05 was set, but the Bonferroni correction was used for pairwise comparisons (adjusted alpha level 0.05/15 = 0.003). The inter-rater agreement of subjective ratings was assessed using Kendall’s *W* statistics. The Kendall’s *W* statistics was interpreted as poor, < 0.20; fair, 0.20–0.40; moderate, 0.40–0.60; good, 0.60–0.80; and excellent, ≥ 0.80 [41]. The post hoc power calculation using sample size and obtained SNR and CNR values, and overall quality ratings in our study obtained 1-beta values of > 0.99, when alpha level was 0.05, indicating high statistical efficiency [42].

## Results

### Participant Characteristics and Radiation Dose

We screened 108 participants and finally included 47 and 47 participants in FD and LD groups, respectively (Fig. 1). The participant demographic characteristics and radiation dose of abdominal CT angiography using FD and LD protocols are summarized in Table 2. The difference in gender, age, and body index is not found between these two groups (all *P* > 0.05). The LD protocol significantly reduced radiation dose compared to FD protocol in terms of volume CT dose index, dose-length product, size-specific dose estimate, and effective dose (all *P* < 0.001).

**Table 2:** Participant characteristics and radiation dose

Characteristics	Full dose (*N* = 47)	Low dose (*N* = 47)	*P* value
Demographic			
Gender, *n* (%)			> 0.999
Male	34 (72)	34 (72)	
Female	13 (28)	13 (28)	
Age, year, mean ± SD, median (IQR)	63.98 ± 10.40, 64.00 (57.00, 71.00)	63.70 ± 10.87, 63.00 (58.00, 71.00)	0.900
Body mass index, kg/m^2^, mean ± SD, median (IQR)	22.42 ± 2.93, 22.04 (19.73, 24.57)	22.16 ± 2.98, 21.88 (19.83, 24.46)	0.666
Radiation dose			
Effective tube current, mAs, mean ± SD, median (IQR)	65.70 ± 14.52, 66.00 (53.00, 76.50)	41.53 ± 9.03, 40.00 (35.00, 48.00)	< 0.001
Volume CT dose index, mGy, mean ± SD, median (IQR)	5.27 ± 1.17, 5.24 (4.28, 6.15)	3.33 ± 0.73, 3.26 (2.80, 3.88)	< 0.001
Dose-length product, mGy·cm, mean ± SD, median (IQR)	155.65 ± 40.79, 147.00 (124.50, 184.00)	95.83 ± 28.68, 91.50 (74.00, 112.00)	< 0.001
Size-specific dose estimates, mGy, mean ± SD, median (IQR)	7.47 ± 1.12, 7.31 (6.51, 8.21)	4.74 ± 0.68, 4.66 (4.25, 5.20)	< 0.001
Effective dose, mSv, mean ± SD, median (IQR)	2.33 ± 0.61, 2.21 (1.87, 2.76)	1.44 ± 0.43, 1.30 (1.11, 1.68)	< 0.001

*SD*, standard deviation; *IQR*, interquartile range

### Objective Image Quality

**Fig. 2:**
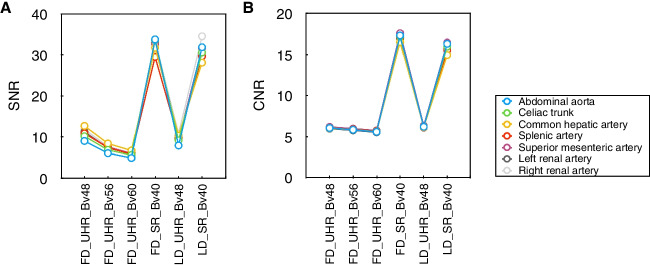
Objective image quality. **A** SNR values of seven arteries. **B** CNR values of seven arteries

The SNR and CNR values are presented in Table 3 and Fig. 2. The SNR and CNR values are significantly higher in 40-keV VMIs than UHR images (all *P* < 0.001). The differences of SNR and CNR values are not found neither between FD_SR_Bv40 and LD_SR_Bv40 images (all *P* ≥ 0.089), nor between FD_UHR_Bv48 and LD_UHR_Bv48 images (all *P* ≥ 0.006). The results for all paired comparisons are summarized (Supplementary Table S2).

**Table 3:** Objective image quality

Metrics, mean ± SD	FD_UHR_Bv48	FD_UHR_Bv56	FD_UHR_Bv60	FD_SR_Bv40	LD_UHR_Bv48	LD_ SR_Bv40
SNR						
Abdominal aorta	9.09 ± 2.01^*#^	6.13 ± 1.38^*#^	4.96 ± 1.09^*#^	33.71 ± 9.29	7.99 ± 1.75^*#^	31.84 ± 9.15
Celiac trunk	10.24 ± 3.51^*#^	6.93 ± 2.20^*#^	5.67 ± 1.74^*#^	33.55 ± 16.51	9.24 ± 2.16^*#^	30.56 ± 20.22
Common hepatic artery	12.74 ± 7.02^*#^	8.48 ± 4.63^*#^	6.77 ± 2.81^*#^	33.73 ± 25.76	10.41 ± 4.58^*#^	28.08 ± 18.89
Splenic artery	10.93 ± 4.28^*#^	7.48 ± 2.47^*#^	5.90 ± 1.90^*#^	29.48 ± 12.79	9.59 ± 2.97^*#^	29.62 ± 12.65
Superior mesenteric artery	10.89 ± 3.75^*#^	7.61 ± 2.58^*#^	6.07 ± 2.00^*#^	32.61 ± 13.81	9.43 ± 2.44^*#^	31.60 ± 14.78
Left renal artery	11.30 ± 3.82^*#^	7.40 ± 2.45^*#^	5.99 ± 2.01^*#^	32.97 ± 18.79	9.88 ± 3.04^*#^	29.73 ± 23.40
Right renal artery	11.89 ± 4.06^*#^	7.79 ± 2.90^*#^	6.11 ± 2.34^*#^	33.42 ± 19.77	10.89 ± 5.64^*#^	34.45 ± 38.77
CNR						
Abdominal aorta	6.07 ± 1.59^*#^	5.86 ± 1.64^*#^	5.61 ± 1.63^*#^	17.29 ± 4.84	6.22 ± 1.69^*#^	16.29 ± 4.54
Celiac trunk	6.09 ± 1.67^*#^	5.87 ± 1.67^*#^	5.64 ± 1.67^*#^	17.19 ± 4.79	6.24 ± 1.70^*#^	15.95 ± 4.32
Common hepatic artery	6.04 ± 1.62^*#^	5.86 ± 1.69^*#^	5.61 ± 1.68^*#^	16.50 ± 4.61	6.17 ± 1.88^*#^	14.92 ± 4.33
Splenic artery	6.08 ± 1.60^*#^	5.92 ± 1.63^*#^	5.71 ± 1.64^*#^	17.06 ± 5.02	6.14 ± 1.88^*#^	15.51 ± 4.42
Superior mesenteric artery	6.21 ± 1.67^*#^	6.00 ± 1.65^*#^	5.77 ± 1.66^*#^	17.56 ± 5.19	6.36 ± 1.78^*#^	16.43 ± 4.51
Left renal artery	5.99 ± 1.66^*#^	5.79 ± 1.68^*#^	5.55 ± 1.69^*#^	16.97 ± 4.76	6.12 ± 1.59^*#^	15.75 ± 4.23
Right renal artery	6.02 ± 1.63^*#^	5.78 ± 1.68^*#^	5.54 ± 1.67^*#^	16.50 ± 4.69	6.19 ± 1.64^*#^	15.64 ± 5.71

*CNR*, contrast-to-noise ratio; *FD*, full dose; *LD*, low dose; *SD*, standard deviation; *SNR*, signal-to-noise ratio; *SR*, standard-reconstruction; *UHR*, ultra-high-resolution

^*^For significantly different compared to SD_SR_Bv40

^#^For significantly different compared to LD_SR_Bv40

The adjusted alpha level using Bonferroni correction is 0.05/15 = 0.003

### Subjective Image Quality

**Fig. 3:**
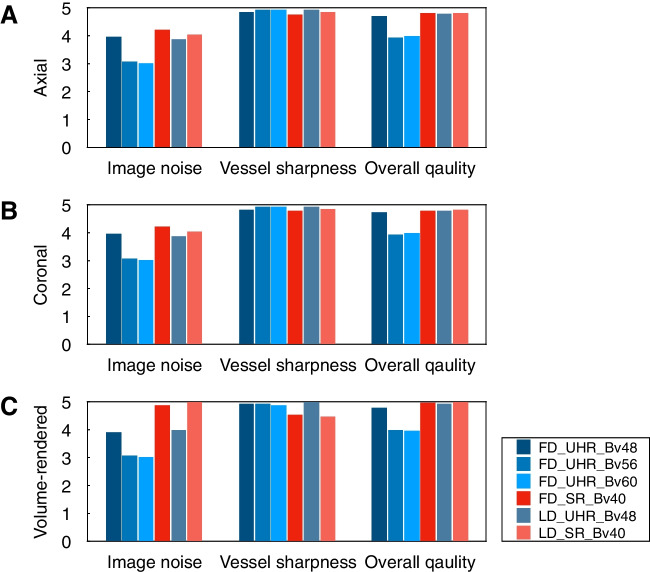
Subjective image quality. The ratings of image noise, vessel sharpness, and overall quality for **A** axial, **B** coronal, and **C** volume-rendered images, respectively, among six series of images

The subjective assessment for image quality is presented in Table 4 and Fig. 3. The subjective ratings showed good to excellent agreement (Kendall’s *W* statistics, 0.77–1.00). For image noise, the FD_SR_Bv40 images presented higher ratings than all four UHR images regardless of axial, cornel, and volume-rendered images (all *P* < 0.001). The LD_SR_Bv40 images showed higher ratings than all four UHR images (all *P* < 0.001), except for cornel and volume-rendered FD_UHR_Bv48 images (*P* = 0.078 and *P* = 0.040). For vessel sharpness, the FD_UHR_Bv56 and FD_UHR_Bv60 images presented higher ratings than FD_SR_Bv40 images regardless of axial, cornel, and volume-rendered images (all *P* ≤ 0.002). The FD_UHR_Bv56 and FD_UHR_Bv60 images showed higher ratings than LD_SR_Bv40 images in volume-rendered images (all *P* ≤ 0.001) but not axial and coronal images (all *P* ≥ 0.017). For overall quality, the FD_UHR_Bv56 and FD_UHR_Bv60 images were rated lower than other four series of images regardless of axial, cornel, and volume-rendered images (all *P* ≤ 0.001). The FD_SR_Bv40, FD_UHR_Bv48, LD_SR_Bv40, and LD_UHR_Bv48 did not show significant difference between each other in axial and cornel images (all *P* ≥ 0.038), while FD_UHR_Bv48 images were rated lower than the other three series of images in volume-rendered images (all *P* ≤ 0.002).

**Table 4:** Subjective image quality and artery visibility

Metrics, mean ± SD (range)	FD_UHR_Bv48	FD_UHR_Bv56	FD_UHR_Bv60	FD_SR_Bv40	LD_UHR_Bv48	LD_SR_Bv40
Axial						
Image noise	3.97 ± 0.21 (3–5)	3.09 ± 0.28 (3–4)	3.02 ± 0.14 (3–4)	4.23 ± 0.46 (3–5)	3.87 ± 0.36 (3–5)	4.04 ± 0.43 (3–5)
Vessel sharpness	4.84 ± 0.36 (4–5)	4.94 ± 0.23 (4–5)	4.94 ± 0.25 (4–5)	4.78 ± 0.42 (4–5)	4.95 ± 0.22 (4–5)	4.87 ± 0.34 (4–5)
Overall quality	4.72 ± 0.46 (3–5)	3.94 ± 0.23 (3–4)	3.99 ± 0.19 (3–5)	4.82 ± 0.38 (4–5)	4.79 ± 0.41 (4–5)	4.83 ± 0.38 (4–5)
Celiac trunk	5.00 ± 0.00 (5–5)	5.00 ± 0.00 (5–5)	5.00 ± 0.00 (5–5)	5.00 ± 0.00 (5–5)	5.00 ± 0.00 (5–5)	5.00 ± 0.00 (5–5)
Common hepatic artery	5.00 ± 0.00 (5–5)	5.00 ± 0.00 (5–5)	5.00 ± 0.00 (5–5)	5.00 ± 0.00 (5–5)	5.00 ± 0.00 (5–5)	5.00 ± 0.00 (5–5)
Hepatic proper artery	4.60 ± 0.53 (3–5)	4.60 ± 0.55 (3–5)	4.60 ± 0.55 (3–5)	4.70 ± 0.46 (4–5)	4.59 ± 0.52 (3–5)	4.60 ± 0.52 (3–5)
Splenic artery	4.79 ± 0.47 (3–5)	4.79 ± 0.47 (3–5)	4.77 ± 0.48 (3–5)	4.84 ± 0.39 (3–5)	4.74 ± 0.69 (1–5)	4.82 ± 0.65 (1–5)
Left gastric artery	4.87 ± 0.38 (3–5)	4.88 ± 0.35 (3–5)	4.87 ± 0.38 (3–5)	4.88 ± 0.33 (4–5)	4.88 ± 0.39 (3–5)	4.83 ± 0.43 (3–5)
Gastroduodenal artery	4.93 ± 0.26 (4–5)	4.92 ± 0.27 (4–5)	4.94 ± 0.25 (4–5)	4.96 ± 0.19 (4–5)	4.91 ± 0.33 (3–5)	4.94 ± 0.25 (4–5)
Superior mesenteric artery	4.90 ± 0.30 (4–5)	4.91 ± 0.29 (4–5)	4.90 ± 0.30 (4–5)	4.90 ± 0.30 (4–5)	4.99 ± 0.12 (4–5)	4.99 ± 0.12 (4–5)
Left renal artery	4.89 ± 0.40 (3–5)	4.89 ± 0.38 (3–5)	4.87 ± 0.42 (3–5)	4.87 ± 0.36 (3–5)	4.85 ± 0.36 (4–5)	4.83 ± 0.38 (4–5)
Right renal artery	4.85 ± 0.46 (3–5)	4.85 ± 0.45 (3–5)	4.83 ± 0.48 (3–5)	4.84 ± 0.38 (3–5)	4.85 ± 0.36 (4–5)	4.83 ± 0.38 (4–5)
Coronal						
Image noise	3.97 ± 0.21 (3–5)	3.09 ± 0.28 (3–4)	3.03 ± 0.17 (3–4)	4.23 ± 0.47 (3–5)	3.88 ± 0.35 (3–5)	4.06 ± 0.44 (3–5)
Vessel sharpness	4.84 ± 0.37 (4–5)	4.94 ± 0.23 (4–5)	4.93 ± 0.28 (3–5)	4.79 ± 0.42 (3–5)	4.95 ± 0.22 (4–5)	4.86 ± 0.35 (4–5)
Overall quality	4.73 ± 0.46 (3–5)	3.94 ± 0.29 (3–5)	4.01 ± 0.22 (3–5)	4.81 ± 0.39 (4–5)	4.80 ± 0.40 (4–5)	4.83 ± 0.38 (4–5)
Celiac trunk	5.00 ± 0.00 (5–5)	5.00 ± 0.00 (5–5)	5.00 ± 0.00 (5–5)	5.00 ± 0.00 (5–5)	4.99 ± 0.12 (4–5)	5.00 ± 0.00 (5–5)
Common hepatic artery	5.00 ± 0.00 (5–5)	5.00 ± 0.00 (5–5)	5.00 ± 0.00 (5–5)	5.00 ± 0.00 (5–5)	5.00 ± 0.00 (5–5)	5.00 ± 0.00 (5–5)
Hepatic proper artery	4.60 ± 0.53 (3–5)	4.60 ± 0.55 (3–5)	4.59 ± 0.56 (3–5)	4.70 ± 0.46 (4–5)	4.57 ± 0.52 (3–5)	4.58 ± 0.54 (3–5)
Splenic artery	4.80 ± 0.45 (3–5)	4.79 ± 0.47 (3–5)	4.79 ± 0.47 (3–5)	4.84 ± 0.38 (3–5)	4.79 ± 0.67 (1–5)	4.82 ± 0.65 (1–5)
Left gastric artery	4.89 ± 0.32 (4–5)	4.89 ± 0.34 (3–5)	4.87 ± 0.38 (3–5)	4.88 ± 0.33 (4–5)	4.83 ± 0.43 (3–5)	4.84 ± 0.42 (3–5)
Gastroduodenal artery	4.93 ± 0.26 (4–5)	4.91 ± 0.28 (4–5)	4.94 ± 0.25 (4–5)	4.96 ± 0.19 (4–5)	4.92 ± 0.32 (3–5)	4.95 ± 0.22 (4–5)
Superior mesenteric artery	4.90 ± 0.30 (4–5)	4.91 ± 0.29 (4–5)	4.90 ± 0.30 (4–5)	4.90 ± 0.30 (4–5)	4.99 ± 0.12 (4–5)	4.99 ± 0.12 (4–5)
Left renal artery	4.89 ± 0.37 (3–5)	4.89 ± 0.38 (3–5)	4.87 ± 0.42 (3–5)	4.87 ± 0.36 (3–5)	4.85 ± 0.36 (4–5)	4.83 ± 0.38 (4–5)
Right renal artery	4.86 ± 0.44 (3–5)	4.85 ± 0.45 (3–5)	4.83 ± 0.48 (3–5)	4.84 ± 0.38 (3–5)	4.85 ± 0.36 (4–5)	4.83 ± 0.38 (4–5)
Volume-rendered images						
Image noise	3.91 ± 0.31 (3–5)	3.09 ± 0.28 (3–4)	3.03 ± 0.24 (2–4)	4.89 ± 0.32 (4–5)	3.98 ± 0.14 (3–4)	4.98 ± 0.14 (4–5)
Vessel sharpness	4.94 ± 0.32 (3–5)	4.92 ± 0.34 (3–5)	4.88 ± 0.42 (3–5)	4.54 ± 0.54 (3–5)	5.00 ± 0.00 (5–5)	4.49 ± 0.50 (4–5)
Overall quality	4.80 ± 0.45 (3–5)	3.99 ± 0.30 (3–5)	3.95 ± 0.30 (3–5)	4.96 ± 0.19 (4–5)	4.94 ± 0.25 (4–5)	5.00 ± 0.00 (5–5)
Celiac trunk	5.00 ± 0.00 (5–5)	5.00 ± 0.00 (5–5)	5.00 ± 0.00 (5–5)	5.00 ± 0.00 (5–5)	5.00 ± 0.00 (5–5)	5.00 ± 0.00 (5–5)
Common hepatic artery	4.96 ± 0.31 (2–5)	4.92 ± 0.46 (2–5)	4.94 ± 0.43 (2–5)	4.97 ± 0.17 (4–5)	4.99 ± 0.17 (3–5)	4.98 ± 0.19 (3–5)
Hepatic proper artery	4.50 ± 0.63 (2–5)	4.47 ± 0.76 (1–5)	4.45 ± 0.77 (2–5)	4.58 ± 0.52 (3–5)	4.43 ± 0.66 (2–5)	4.35 ± 0.78 (1–5)
Splenic artery	4.82 ± 0.59 (2–5)	4.83 ± 0.56 (2–5)	4.81 ± 0.57 (2–5)	4.86 ± 0.35 (4–5)	4.78 ± 0.72 (1–5)	4.76 ± 0.75 (1–5)
Left gastric artery	4.57 ± 0.92 (1–5)	4.60 ± 0.91 (1–5)	4.59 ± 0.93 (1–5)	4.77 ± 0.68 (1–5)	4.39 ± 0.97 (1–5)	4.52 ± 0.83 (1–5)
Gastroduodenal artery	4.74 ± 0.79 (1–5)	4.75 ± 0.78 (1–5)	4.72 ± 0.79 (1–5)	4.79 ± 0.57 (2–5)	4.76 ± 0.73 (1–5)	4.77 ± 0.71 (1–5)
Superior mesenteric artery	4.82 ± 0.47 (2–5)	4.84 ± 0.46 (2–5)	4.84 ± 0.44 (3–5)	4.85 ± 0.38 (3–5)	4.87 ± 0.34 (4–5)	4.84 ± 0.42 (3–5)
Left renal artery	4.60 ± 0.63 (2–5)	4.60 ± 0.68 (1–5)	4.52 ± 0.67 (2–5)	4.35 ± 0.55 (3–5)	4.61 ± 0.61 (3–5)	4.40 ± 0.64 (3–5)
Right renal artery	4.55 ± 0.76 (1–5)	4.54 ± 0.77 (1–5)	4.49 ± 0.77 (1–5)	4.31 ± 0.68 (2–5)	4.55 ± 0.58 (3–5)	4.36 ± 0.66 (3–5)

*FD*, full dose; *LD*, low dose; *SD*, standard deviation; *SR*, standard-reconstruction; *UHR*, ultra-high resolution

^*^For significantly different compared to SD_SR_Bv40

^#^For significantly different compared to LD_SR_Bv40

The adjusted alpha level using Bonferroni correction is 0.05/15 = 0.003

### Artery Visibility

**Fig. 4:**
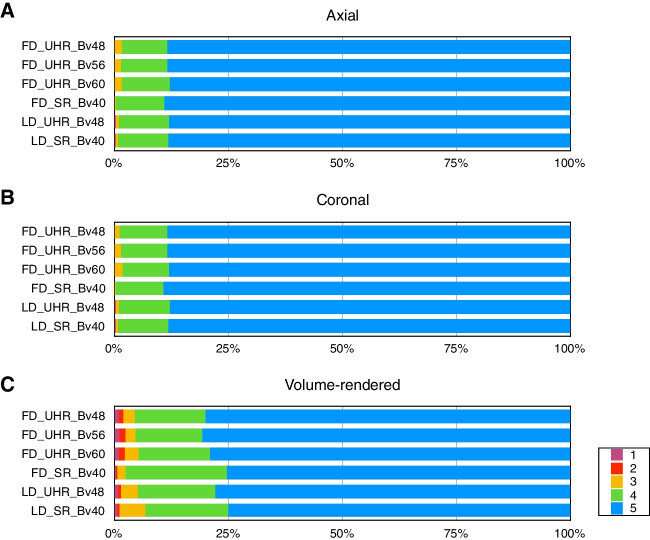
Artery visibility. The artery visibility for **A** axial, **B** coronal, and **C** volume-rendered images, respectively, among six series of images

**Fig. 5:**
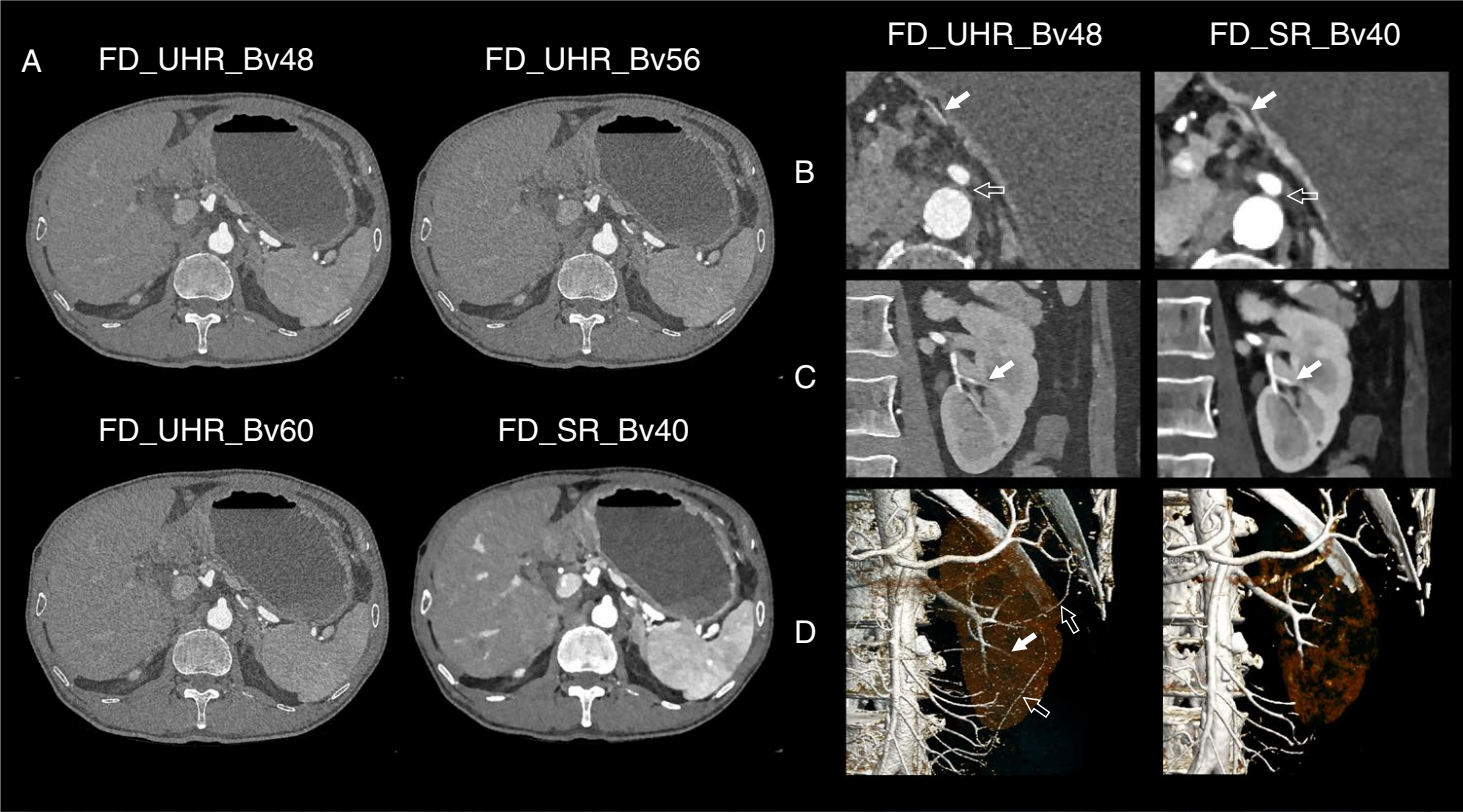
Representative example of two participants who both underwent full-dose scan. **A** A 71-year man with a BMI of 19.7 kg/m^2^ underwent abdominal CTA using a FD protocol with CTDIvol, DLP, SSDE, and effective dose of 6.23 mGy, 212.0 mGy·cm, 9.06 mGy, and 3.18 mSv, respectively. The data were reconstructed into FD_UHR_Bv48, FD_UHR_Bv56, FD_UHR_Bv60, FD_SR_Bv40 images, respectively. The UHR images have higher image noise, but better vessel sharpness. The overall quality of FD_UHR_Bv48, and FD_SR_Bv40 images are comparable. The FD_UHR_Bv56 and FD_UHR_Bv60 images presented better vessel sharpness than but were damaged by higher image noise. **B**–**D** Another 57-year woman with a BMI of 20.8 kg/m.^2^ underwent abdominal CTA using a FD protocol with CTDIvol, DLP, SSDE, and effective dose of 4.06 mGy, 111.0 mGy·cm, 6.25 mGy, and 1.67 mSv, respectively. The data were reconstructed into FD_UHR_Bv48, and FD_SR_Bv40 images, respectively. **B** The left gastric artery is only visible in axial FD_UHR_Bv48 images (solid arrow), and the soft tissue between the abdominal aorta and celiac trunk were better depicted (open arrow). **C** There were more visible vascular segments of left renal artery in coronal FD_UHR_Bv48 images than FD_SR_Bv40 images (solid arrow). **D** The left renal artery can be better visualized to the more distal vascular segments in volume-rendered FD_UHR_Bv48 images than FD_SR_Bv40 images (solid arrow). The volume-rendered FD_UHR_Bv48 images allowed more visible vascular segments of superior mesenteric artery (open arrow)

**Fig. 6:**
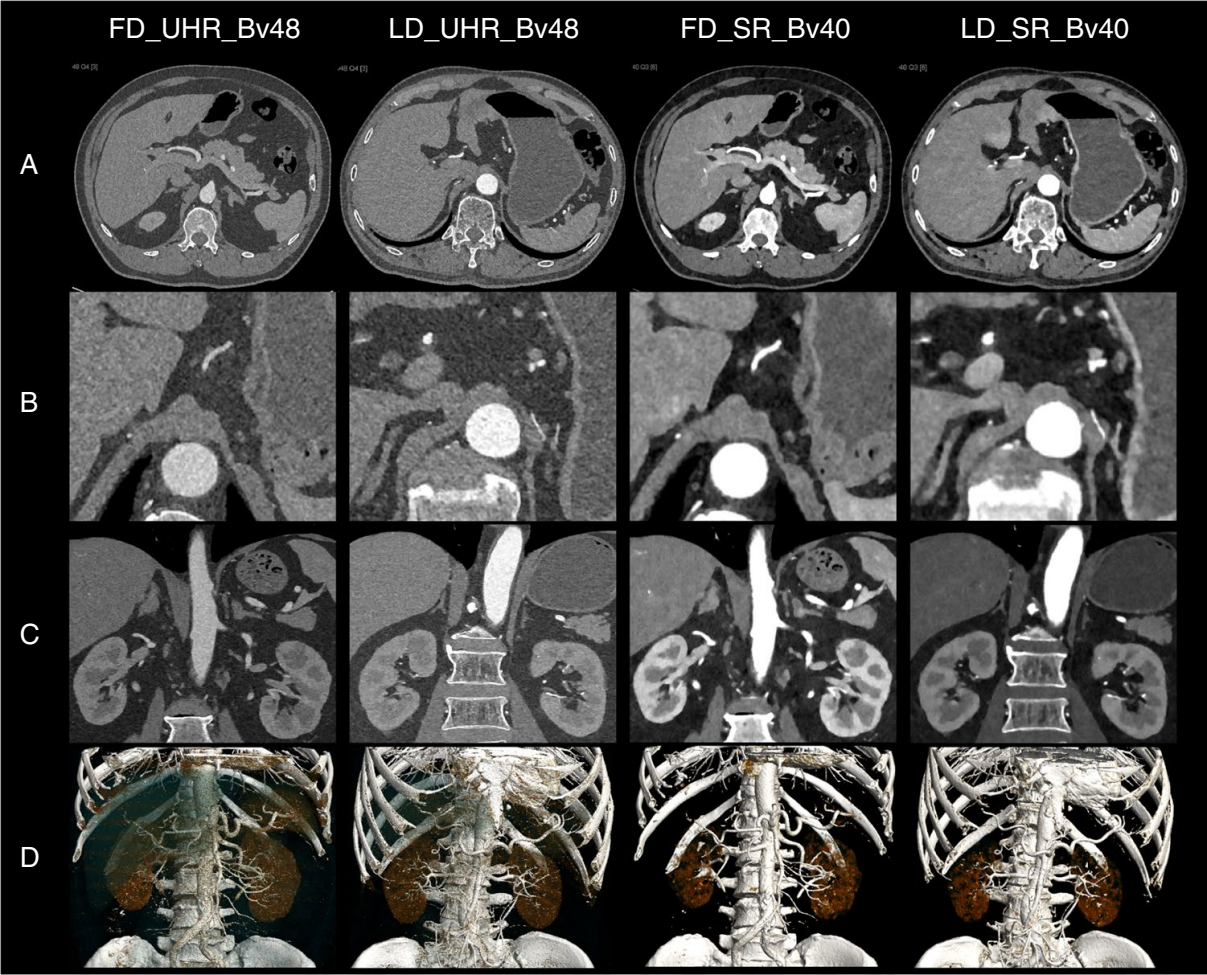
Representative example of two participants who underwent full-dose and low-dose scan. A 61-year man with a BMI of 29.8 kg/m^2^ underwent abdominal CTA using a FD protocol with CTDIvol, DLP, SSDE, and effective dose of 8.75 mGy, 245.0 mGy·cm, 10.50 mGy, and 3.68 mSv, respectively (the first and the third column). Another 62-year man with a BMI of 23.2 kg/m^2^ underwent abdominal CTA using a LD protocol with CTDIvol, DLP, SSDE, and effective dose of 3.60 mGy, 106.0 mGy·cm, 5.05 mGy, and 1.59 mSv, respectively (the second and the fourth column). The data were reconstructed into FD_UHR_Bv48, LD_UHR_Bv48, FD_SR_Bv40, LD_SR_Bv40 images, respectively. **A** The overall quality of FD and LD images were comparable. **B**–**D** The artery visibility of FD and LD images were comparable regardless of axial, coronal and volume-rendered images. The UHR images allowed more vascular segments and thinner image slice but with higher image noise. The SR images showed better contrast but lower vessel sharpness and thicker image slice

The artery visibility is presented in Table 4 and Fig. 4. Most of the evaluated vessels were rated at least 5 (84.90%, 19,393/22842), suggesting that all vascular segments from the artery trunk to the subsegmental peripheral artery were clearly visible. The arteries with a rating of 5 for the axial images (88.42%, 6732/7614) and coronal images (88.49%, 6738.7614) were more than those in VR images (80.06%, 6096/7614). Almost all the evaluated arteries were rated at least 3 (99.44%, 22,714/22842), indicating that the arteries are visible from the trunk to at least half of the vascular segments. The arteries with a rating at least 3 for each series of images were FD_UHR_Bv48 (99.37%, 3783/3807), FD_UHR_Bv56 (99.21%, 3777/3807), FD_UHR_Bv60 (99.24%, 3778/3807), FD_SR_Bv40 (99.79%, 3799/3807), LD_UHR_Bv48 (99.40%, 3784/3807), and LD_SR_Bv40 (99.47%, 3787/3807). The difference in artery visibility were not found in most comparisons (95.3%, 386/405). The results for all paired comparisons are summarized (Supplementary Table S3). Representative examples are presented in Figs. 5 and 6.

## Discussion

Our study found that SR images of low-keV VMI and UHR images with a kernel of Bv48 are preferred for visualization of abdominal arteries. The sharper kernels of Bv56 and Bv60 led to higher image noise that damaged overall quality and therefore might not be suitable for visualization of abdominal arteries. Furthermore, the SR and UHR images have potential to provide similar image quality and artery visibility with a reduced radiation dose. We believed that the 1-mm LD_40keV_VMI can serve as an optimized choice for daily assessment of abdominal arteries, while the 0.2-mm LD_UHR_Bv48 images can be used as an alternative with similar image quality and artery visibility when thinner slice image is necessary.

In our study, the VMI at 40-keV reconstructed using raw data acquired by UHR scan has been used as the reference standard for comparison. Dillinger et al. [18] have showed the advantage of PCD-CT to generate VMI to present the abdominal arteries with optimal image quality and vessel contrast regardless of vessel size. Hennes et al. [19] and Higashigaito et al. [17] further demonstrated the potential of VMI from PCD-CT in reduction of radiation dose and iodinated contrast media. Therefore, we chose the low-keV VMI as the state-of-art reference imaging modality for CTA of abdominal arteries to test whether the UHR images can provide further improvements in image quality and artery visibility. On the other hand, phantom study of abdomen arteries has showed that UHR acquisition with sharper reconstruction kernels allow a significant noise reduction in comparison to acquisitions using standard pixels at the same resolution and noise level [43]. The phantom study in coronary arteries further demonstrated the improved stenosis quantification and reduced blooming artifacts with UHR acquisition compared to standard resolution acquisition [26]. Moreover, the cadaveric specimen study in CTA of femoral arteries indicated that the UHR acquisition in combination with ultrasharp reconstruction kernels can provide better image quality than standard resolution acquisitions [28]. However, it is not possible to directly compare the images acquired using UHR and standard resolution acquisitions in our study due to the concern of radiation dose. Thus, our study used low-keV VMI reconstructed using raw data from UHR acquisition instead. We believe this selection actually enhanced our study since the low-keV VMI is expected to better visualize the arteries than polychromatic images generated from standard resolution acquisitions.

The previous CTA studies of coronary and femoral arteries recommended UHR acquisition combing with sharp reconstruction kernels higher than Bv60 for better vessel sharpness at the expense of worse image noise [23–27]. It may be a good deal for coronary and femoral arteries but is not suitable for abdominal arteries that already suffering from image noise. These sharper kernels did provide better vessel sharpness in subjective assessment but were not preferred by radiologists due to the increased image noise. The low-keV VMI, although with lower vessel sharpness, benefited by the better image contrast and noise, can better present the abdominal arteries than UHR images with these sharp kernels. Our study suggested that a not-so-sharp kernel of Bv48 is a better choice for abdominal arteries than sharper ones of Bv56 and Bv60. The UHR images with a kernel of Bv48 had comparable image quality as low-keV VMI and further allowed thinner slice thickness for evaluation of small segments of abdominal arteries. Indeed, the UHR images with Bv48 presented more small segments of abdominal arteries that was invisible in low-keV VMI. The better visualization of the small segments of abdominal arteries can be directly translated into higher accuracy in tumor staging and optimized operation planning [44].

Our study presented the potential of UHR images with Bv48 and low-keV VMI in radiation dose reduction. The techniques for radiation dose reduction includes adjustment of tube current and/or voltage, automatic control of image quality, high pitch scans, new reconstruction algorithms, etc. Our study was benefited by the development of the photon-counting detector, which allows adequate image quality with an additional radiation reduction [13–15]. In our study, the objective measurements did not find significant difference between FD and LD protocol neither in UHR images with Bv48 nor in low-keV VMI. The subjective assessment also supported these results by comparable ratings in image quality and artery visibility. Our study further provided evidence that the UHR acquisition allows lower radiation dose for patients in clinical practice. The UHR scans allows lower images noise and better image quality because of the small pixel effect [43, 45, 46], and therefore can realize comparable image quality with lower radiation dose. In previous phantom and cadaveric specimen studies, the UHR acquisition with sharp reconstruction kernel was expected to reduce radiation dose compared to standard resolution acquisitions according to the estimation of decreasing image noise [28, 43, 45, 46]. The percentage of radiation dose in our study was not as large as their estimations. It can be attribute to the reference standard used in the above-mentioned studies that were images reconstructed using standard resolution acquisitions. In contrast, our study chose low-keV VMI that reconstructed using UHR acquisitions as reference. It is expected to have optimal image quality and artery visibility than those reconstructed using standard resolution acquisitions, not allowing further radiation dose reduction.

We have to address several limitations of our study. First, this is a single-center study for underlying benefits of UHR in abdominal CT angiography with a relatively small sample size and with a small proportion of women. Our study design and patient availability limit the sample size of our study [47]. It is necessary to validate our findings, and further evaluate the potential of diagnostic improvement. Second, the VMIs were reconstructed using data from UHR scan mode. Further studies are needed to compare the image quality of UHR images and VMIs that generated using data from different scan modes. Third, the visualization of the abdominal arteries was only compared among CT images. We have not compared them to the excised specimens. However, it is believed that the optimized visualization of the segments of arteries can better guide operation planning. Fourth, our study applied a five-point Likert scale for image quality and artery visibility assessment. A pairwise comparison method is more reliable for subjective image assessment and may serve as a better tool for studies with smaller variation and more difficult comparisons [48]. Finally, we did not meter the reading time for six series of images with different slice thickness and corresponding number of slices. It is expected to be time-consuming and labor-intensive for radiologists to interpret numerous thin slice images of a large scan range.

In conclusion, we considered that 1-mm SR image of VMI at 40-keV is superior to 0.2-mm UHR regardless of which kernel is used to visualize abdominal arteries, while 0.2-mm UHR image using a relatively smooth kernel may allow similar image quality and artery visibility when thinner slice image is warranted.

## Supplementary Information

Below is the link to the electronic supplementary material.

Supplementary file1 (DOCX 24.3 MB)

## Data Availability

The data that support the findings of this study are available from the corresponding author, upon reasonable request.

## Abbreviations

UHR: Ultra-high-resolution
PCD-CT: Photon-counting detector computed tomography
keV: Kiloelectron volt
VMI: Virtual monoenergetic imaging
FD: Full dose
LD: Low-dose
SNR: Signal-to-noise ratio
CNR: Contrast-to-noise ratio
SD: Standard deviation
IQR: Interquartile range
SR: Standard-reconstruction
QIR: Quantum iterative reconstruction
ROI: Region of interest

## Declarations

### Overlapping Cohort

The abstract of this study entitled “Does ultra-high-resolution photon-counting detector CT benefit visualization of abdominal arteries? A comparison to standard-reconstruction” (C-22800) has been accepted as a digital poster, EPOS Radiologist (scientific), on European Congress of Radiology 2024 (https://doi.org/10.26044/ecr2024/C-22800). The presenting author of this abstract is Dr. Jingyu Zhong.
