# Supplementary material for: Ultra-High-Resolution Photon-Counting Detector CT Benefits Visualization of Abdominal Arteries: A Comparison to Standard-Reconstruction
Source: J Imaging Inform Med. 2024 Oct 25;38(3):1891–903. doi: 10.1007/s10278-024-01232-5 (PMC12092866; doi:10.1007/s10278-024-01232-5)
Supplement: Supplementary file 1 — Supplementary file1 (DOCX 24.3 MB) [file 10278_2024_1232_MOESM1_ESM.docx]

**Supplementary Material**

**Title:** Ultra-high-resolution photon-counting detector CT benefits visualization of abdominal arteries: A comparison to standard-reconstruction

**List of Supplementary Material**

Supplementary Note S1 Objective image assessment

Supplementary Note S2 Subjective image assessment

Supplementary Table S1 Results of preliminary study

Supplementary Table S1 Results of all paired comparisons of objective image assessment

Supplementary Table S2 Results of all paired comparisons of subjective image assessment

**Supplementary Note S1 Objective image assessment**

The objective image quality assessment was performed by a radiologist with 5 years of experience using the workstation (Syngo.Via, version VB60, Siemens Healthineers) with default tools. The regions of interest (ROIs) were put on seven arteries (abdominal aorta, celiac trunk, common hepatic artery, splenic artery, superior mesenteric artery, left renal artery, and right renal artery), psoas muscle, and subcutaneous fat, for CT number values and corresponding standard deviation (SD) values. The circular ROIs with a diameter of 3 to 15 mm were put on axial slices that present the arteries, covering the vascular lumen as much as possible while avoiding to touch vascular walls, calcification, thrombus, or artifacts. Then, the ROIs were copied and pasted to other series of images. The SD values of homogeneous anterior abdominal subcutaneous fat tissues at the third lumbar vertebra level was defined as the background noise. The CT number values of psoas muscle were recorded. Each HU and SD value was calculated by averaging the measurements of three consecutive axial image slices.

Here are examples of the measurement of abdominal aorta, psoas muscle, and subcutaneous fat.

Note the example images are not included in current study. The ROIs are just diagrams.


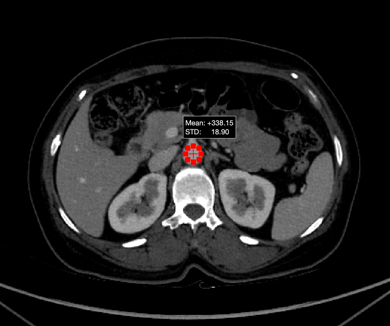

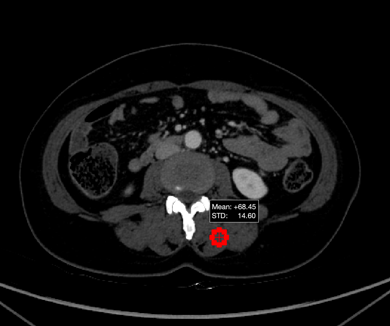

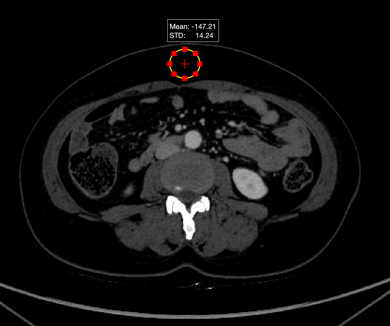


The signal-to-noise ratio (SNR) was calculated as SNR_artery_ = HU_artery_ / SD_artery_, where the HU_artery_ is the mean CT value of the interested artery ROI, SD_artery_ is the standard deviation value of the interested artery ROI.

The contrast-to-noise ratio (CNR) was calculated as CNR_artery_ = (HU_artery_ - HU_muscle_) / SD_fat_, where the HU_artery_ is the mean CT value of the interested artery ROI, HU_muscle_ is the mean CT value of the psoas muscle ROI, SD_fat_ is the standard deviation value of the interested subcutaneous fat ROI.

**Supplementary Note S2 Subjective image assessment**

The subjective image quality assessment was performed by three radiologists with 5, 5, and 6 years of experience, respectively. The experience was defined as the total experience of reading CT including the experience of training and post-training. Each reader interpreted about 10,000 CT examinations each year in our institution, in which about 3,000 were abdominopelvic CT examinations.

The subjective image quality assessment was performed in the reading room using medical monitors with daily settings. All rating was performed using monitors (MDCC-4430, BRACO Co. Ltd., Belgium) with the same settings (system firmware version, v1.02.00; native resolution/pixel format 2560 × 1600; physical size, 654 × 409 mm; luminance 600 cd/m^2^) employed for daily image interpretation at the reading room (Ambient Light, 10 lx). The images were presented to the readers in a random fashion blinded to the reconstruction parameters, with a default window width of 700 HU and window level of 80 HU. The readers were allowed to adjust the window width and level and viewing distance as they preferred and had no time limits to complete the image review.

**1. Subjective image quality assessment**

The image quality was assessed in terms of image noise, vessel sharpness, and overall quality. A five-point Likert scale was used: 1, unacceptable; 2, suboptimal; 3, acceptable; 4, good; and 5, excellent. The definitions of image quality rating for each term are as follows:

| **Rating** | **Image noise** | **Vessel sharpness** | **Overall quality** |
| --- | --- | --- | --- |
| 1 | Unacceptable, severe noise; nondiagnostic | substantial blurring of vessel structures; nondiagnostic | Non-diagnostic; poor delineation of vessel structures and non-diagnostic image quality |
| 3 | Moderate, acceptable noise for diagnosis | Blurring of liver and vessel edges but not affecting diagnostic confidence | Satisfactory, good delineation of vessel structures and diagnostic image quality |
| 5 | Minimal, excellent image with low noise | Excellent quality with sharp liver borders and vessel edges | Excellent; sharp delineation of vessel structures and excellent diagnostic image quality |

Here is an example of six series of images: FD_UHR_Bv48, FD_UHR_Bv56, FD_UHR_Bv60, FD_ SR_Bv40, LD_UHR_Bv48, and LD_SR_Bv40. Note that the reconstruction parameters blinded to the readers during the readout.

**2. Vessel visibility assessment**

The visibility of nine arteries (celiac trunk, common hepatic artery, hepatic proper artery, splenic artery, left gastric artery, gastroduodenal artery, superior mesenteric artery, left renal artery, and right renal artery) were evaluated using axial, coronal, volume rendered images. A five-point Likert scale was defined as follow.

| **Rating** | **Vessel visibility** |
| --- | --- |
| 1 | no vascular segment was clearly visualized |
| 3 | nearly half of the vascular segments were clearly visualized |
| 5 | all vascular segments from the trunk to the subsegmental peripheral artery were clearly visualized |

Here are examples of vessel visibility with different ratings. Note that the reconstruction parameters blinded to the readers during the readout.

**Supplementary Table S1 Results of preliminary study**

| N = 10 | Overall quality | P value | Image noise | P value | Vessel sharpness | P value |
| --- | --- | --- | --- | --- | --- | --- |
| FD_SR_Bv40 | 4.8 ± 0.4, 5 (4-5) | n. a. | 4.3 ± 0.5, 4 (4-5) | n. a. | 4.9 ± 0.3, 5 (4-5) | n. a. |
| LD_UHR_Bv48 | 4.9 ± 0.3, 5 (4-5) | 0.556 | 4.0 ± 0.0, 4 (4-4) | 0.067 | 5.0 ± 0.0, 5 (5-5) | 0.331 |
| LD_UHR_Bv56 | 3.9 ± 0.3, 4 (3-4) | <0.001 | 3.0 ± 0.0, 3 (3-3) | <0.001 | 4.9 ± 0.3, 5 (4-5) | >0.999 |
| LD_UHR_Bv60 | 3.8 ± 0.4, 4 (3-4) | <0.001 | 3.1 ± 0.3, 3 (3-4) | <0.001 | 5.0 ± 0.0, 5 (5-5) | 0.331 |
| LD_SR_Bv40 | 4.8 ± 0.4, 5 (4-5) | >0.999 | 4.2 ± 0.4, 4 (4-5) | 0.628 | 4.6 ± 0.5, 4 (4-5) | 0.135 |

The numbers are presented as mean ± standard deviation, median (range). The P values are compared to the reference standard (FD_SR_Bv40) using Mann-Whitney U test. The alpha level is 0.05. The P values with significance were marked in red. The preliminary study of ten participants in FD and LD groups, respectively, indicated that the LD_UHR_Bv56 and LD_UHR_Bv60 images were not optimal for clinical because of the worse overall quality due to image noise. FD = full dose, LD = low dose, UHR = ultra-high resolution, SR = standard-reconstruction.

**Supplementary Table S2 Results of all paired comparisons of objective image assessment**

| Group 1 | Group 2 | Abdominal aorta | Celiac trunk | Common hepatic artery | Splenic artery | Superior mesenteric artery | Left renal artery | Right renal artery |
| --- | --- | --- | --- | --- | --- | --- | --- | --- |
| SNR |  |  |  |  |  |  |  |  |
| FD_SR_40keV | FD_UHR_Bv48 | <0.001 | <0.001 | <0.001 | <0.001 | <0.001 | <0.001 | <0.001 |
| FD_SR_40keV | FD_UHR_Bv56 | <0.001 | <0.001 | <0.001 | <0.001 | <0.001 | <0.001 | <0.001 |
| FD_SR_40keV | FD_UHR_Bv60 | <0.001 | <0.001 | <0.001 | <0.001 | <0.001 | <0.001 | <0.001 |
| FD_SR_40keV | LD_UHR_Bv48 | <0.001 | <0.001 | <0.001 | <0.001 | <0.001 | <0.001 | <0.001 |
| FD_SR_40keV | LD_SR_40keV | 0.330 | 0.435 | 0.412 | 0.957 | 0.734 | 0.461 | 0.871 |
| LD_SR_40keV | FD_UHR_Bv48 | <0.001 | <0.001 | <0.001 | <0.001 | <0.001 | <0.001 | <0.001 |
| LD_SR_40keV | FD_UHR_Bv56 | <0.001 | <0.001 | <0.001 | <0.001 | <0.001 | <0.001 | <0.001 |
| LD_SR_40keV | FD_UHR_Bv60 | <0.001 | <0.001 | <0.001 | <0.001 | <0.001 | <0.001 | <0.001 |
| LD_SR_40keV | LD_UHR_Bv48 | <0.001 | <0.001 | <0.001 | <0.001 | <0.001 | <0.001 | <0.001 |
| FD_UHR_Bv48 | FD_UHR_Bv56 | <0.001 | <0.001 | 0.001 | <0.001 | <0.001 | <0.001 | <0.001 |
| FD_UHR_Bv48 | FD_UHR_Bv60 | <0.001 | <0.001 | <0.001 | <0.001 | <0.001 | <0.001 | <0.001 |
| FD_UHR_Bv48 | LD_UHR_Bv48 | 0.006 | 0.099 | 0.059 | 0.082 | 0.028 | 0.050 | 0.326 |
| FD_UHR_Bv56 | FD_UHR_Bv60 | <0.001 | 0.003 | 0.034 | 0.001 | 0.002 | 0.003 | 0.003 |
| FD_UHR_Bv56 | LD_UHR_Bv48 | <0.001 | <0.001 | 0.045 | <0.001 | 0.001 | <0.001 | 0.001 |
| CNR |  |  |  |  |  |  |  |  |
| FD_SR_40keV | FD_UHR_Bv48 | <0.001 | <0.001 | <0.001 | <0.001 | <0.001 | <0.001 | <0.001 |
| FD_SR_40keV | FD_UHR_Bv56 | <0.001 | <0.001 | <0.001 | <0.001 | <0.001 | <0.001 | <0.001 |
| FD_SR_40keV | FD_UHR_Bv60 | <0.001 | <0.001 | <0.001 | <0.001 | <0.001 | <0.001 | <0.001 |
| FD_SR_40keV | LD_UHR_Bv48 | <0.001 | <0.001 | <0.001 | <0.001 | <0.001 | <0.001 | <0.001 |
| FD_SR_40keV | LD_SR_40keV | 0.306 | 0.191 | 0.089 | 0.115 | 0.262 | 0.193 | 0.426 |
| LD_SR_40keV | FD_UHR_Bv48 | <0.001 | <0.001 | <0.001 | <0.001 | <0.001 | <0.001 | <0.001 |
| LD_SR_40keV | FD_UHR_Bv56 | <0.001 | <0.001 | <0.001 | <0.001 | <0.001 | <0.001 | <0.001 |
| LD_SR_40keV | FD_UHR_Bv60 | <0.001 | <0.001 | <0.001 | CNR | <0.001 | <0.001 | <0.001 |
| LD_SR_40keV | LD_UHR_Bv48 | <0.001 | <0.001 | <0.001 | <0.001 | <0.001 | <0.001 | <0.001 |
| FD_UHR_Bv48 | FD_UHR_Bv56 | 0.527 | 0.534 | 0.609 | 0.626 | 0.548 | 0.561 | 0.489 |
| FD_UHR_Bv48 | FD_UHR_Bv60 | 0.168 | 0.197 | 0.215 | 0.267 | 0.205 | 0.212 | 0.165 |
| FD_UHR_Bv48 | LD_UHR_Bv48 | 0.663 | 0.673 | 0.712 | 0.872 | 0.676 | 0.683 | 0.599 |
| FD_UHR_Bv56 | FD_UHR_Bv60 | 0.461 | 0.501 | 0.475 | 0.534 | 0.498 | 0.505 | 0.491 |
| FD_UHR_Bv56 | LD_UHR_Bv48 | 0.299 | 0.300 | 0.404 | 0.544 | 0.318 | 0.319 | 0.229 |

CNR = contrast-to-noise ratio, FD = full dose, keV = kiloelectron volt, LD = low dose, SD = standard deviation, SNR = signal-to-noise ratio, UHR = ultra-high resolution, SR = standard-reconstruction. The adjusted alpha level using Bonferroni correction is 0.05/15 = 0.003. The P values with significance were marked in red.

**Supplementary Table S3 Results of all paired comparisons of subjective image assessment**

| Group 1 | Group 2 | Image noise | Vessel sharpness | Overall quality | Celiac trunk | Common hepatic artery | Hepatic proper artery | Splenic artery | Left gastric artery | Gastroduodenal artery | Superior mesenteric artery | Left renal artery | Right renal artery |
| --- | --- | --- | --- | --- | --- | --- | --- | --- | --- | --- | --- | --- | --- |
| Axial |  |  |  |  |  |  |  |  |  |  |  |  |  |
| FD_SR_40keV | FD_UHR_Bv48 | <0.001 | 0.171 | 0.051 | >0.999 | >0.999 | 0.096 | 0.409 | 0.738 | 0.186 | >0.999 | 0.753 | 0.889 |
| FD_SR_40keV | FD_UHR_Bv56 | <0.001 | <0.001 | <0.001 | >0.999 | >0.999 | 0.101 | 0.409 | >0.999 | 0.123 | 0.840 | 0.746 | 0.886 |
| FD_SR_40keV | FD_UHR_Bv60 | <0.001 | <0.001 | <0.001 | >0.999 | >0.999 | 0.101 | 0.223 | 0.866 | 0.274 | >0.999 | 0.878 | 0.783 |
| FD_SR_40keV | LD_UHR_Bv48 | <0.001 | <0.001 | 0.547 | >0.999 | >0.999 | 0.071 | 0.139 | >0.999 | 0.118 | 0.002 | 0.617 | 0.872 |
| FD_SR_40keV | LD_SR_40keV | <0.001 | 0.062 | 0.876 | >0.999 | >0.999 | 0.116 | 0.824 | 0.276 | 0.274 | 0.002 | 0.331 | 0.754 |
| LD_SR_40keV | FD_UHR_Bv48 | 0.078 | 0.614 | 0.036 | >0.999 | >0.999 | 0.910 | 0.674 | 0.465 | 0.813 | 0.002 | 0.220 | 0.672 |
| LD_SR_40keV | FD_UHR_Bv56 | <0.001 | 0.026 | <0.001 | >0.999 | >0.999 | 0.911 | 0.674 | 0.288 | 0.644 | 0.003 | 0.209 | 0.666 |
| LD_SR_40keV | FD_UHR_Bv60 | <0.001 | 0.047 | <0.001 | >0.999 | >0.999 | 0.911 | 0.466 | 0.377 | >0.999 | 0.002 | 0.455 | >0.999 |
| LD_SR_40keV | LD_UHR_Bv48 | <0.001 | 0.013 | 0.448 | >0.999 | >0.999 | 0.819 | 0.287 | 0.309 | 0.537 | >0.999 | 0.627 | 0.627 |
| FD_UHR_Bv48 | FD_UHR_Bv56 | <0.001 | 0.007 | <0.001 | >0.999 | >0.999 | >0.999 | >0.999 | 0.745 | 0.821 | 0.840 | >0.999 | >0.999 |
| FD_UHR_Bv48 | FD_UHR_Bv60 | <0.001 | 0.013 | <0.001 | >0.999 | >0.999 | >0.999 | 0.708 | 0.875 | 0.813 | >0.999 | 0.662 | 0.704 |
| FD_UHR_Bv48 | LD_UHR_Bv48 | 0.004 | 0.003 | 0.173 | >0.999 | >0.999 | 0.910 | 0.422 | 0.757 | 0.686 | 0.002 | 0.432 | >0.999 |
| FD_UHR_Bv56 | FD_UHR_Bv60 | 0.017 | 0.803 | 0.050 | >0.999 | >0.999 | >0.999 | 0.708 | 0.869 | 0.644 | 0.840 | 0.655 | 0.699 |
| FD_UHR_Bv56 | LD_UHR_Bv48 | <0.001 | 0.007 | <0.001 | >0.999 | >0.999 | >0.999 | >0.999 | 0.745 | 0.821 | 0.840 | >0.999 | >0.999 |
| FD_SR_40keV | FD_UHR_Bv48 | <0.001 | 0.608 | <0.001 | >0.999 | >0.999 | 0.911 | 0.619 | 0.876 | 0.537 | 0.002 | 0.760 | 0.672 |
| Coronal |  |  |  |  |  |  |  |  |  |  |  |  |  |
| FD_SR_40keV | FD_UHR_Bv48 | <0.001 | 0.370 | 0.128 | >0.999 | >0.999 | 0.074 | 0.394 | 0.854 | 0.186 | >0.999 | 0.624 | 0.773 |
| FD_SR_40keV | FD_UHR_Bv56 | <0.001 | <0.001 | <0.001 | >0.999 | >0.999 | 0.100 | 0.332 | 0.858 | 0.080 | 0.840 | 0.746 | 0.886 |
| FD_SR_40keV | FD_UHR_Bv60 | <0.001 | 0.002 | <0.001 | >0.999 | >0.999 | 0.064 | 0.332 | 0.866 | 0.274 | >0.999 | 0.878 | 0.783 |
| FD_SR_40keV | LD_UHR_Bv48 | <0.001 | <0.001 | 0.881 | 0.157 | >0.999 | 0.030 | 0.386 | 0.276 | 0.171 | 0.002 | 0.617 | 0.872 |
| FD_SR_40keV | LD_SR_40keV | 0.001 | 0.169 | 0.644 | >0.999 | >0.999 | 0.044 | 0.737 | 0.347 | 0.557 | 0.002 | 0.331 | 0.754 |
| LD_SR_40keV | FD_UHR_Bv48 | 0.040 | 0.621 | 0.049 | >0.999 | >0.999 | 0.824 | 0.749 | 0.268 | 0.455 | 0.002 | 0.154 | 0.562 |
| LD_SR_40keV | FD_UHR_Bv56 | <0.001 | 0.017 | <0.001 | >0.999 | >0.999 | 0.742 | 0.674 | 0.279 | 0.236 | 0.003 | 0.209 | 0.666 |
| LD_SR_40keV | FD_UHR_Bv60 | <0.001 | 0.063 | <0.001 | >0.999 | >0.999 | 0.914 | 0.674 | 0.458 | 0.608 | 0.002 | 0.455 | >0.999 |
| LD_SR_40keV | LD_UHR_Bv48 | <0.001 | 0.008 | 0.541 | 0.157 | >0.999 | 0.911 | 0.653 | 0.889 | 0.383 | >0.999 | 0.627 | 0.627 |
| FD_UHR_Bv48 | FD_UHR_Bv56 | <0.001 | 0.004 | <0.001 | >0.999 | >0.999 | 0.912 | 0.897 | >0.999 | 0.658 | 0.840 | 0.874 | 0.893 |
| FD_UHR_Bv48 | FD_UHR_Bv60 | <0.001 | 0.020 | <0.001 | >0.999 | >0.999 | 0.914 | 0.897 | 0.732 | 0.813 | >0.999 | 0.548 | 0.604 |
| FD_UHR_Bv48 | LD_UHR_Bv48 | 0.007 | 0.002 | 0.169 | 0.157 | >0.999 | 0.736 | 0.836 | 0.209 | 0.837 | 0.002 | 0.328 | 0.882 |
| FD_UHR_Bv56 | FD_UHR_Bv60 | 0.040 | 0.646 | 0.038 | >0.999 | >0.999 | 0.830 | >0.999 | 0.740 | 0.498 | 0.840 | 0.655 | 0.699 |
| FD_UHR_Bv56 | LD_UHR_Bv48 | <0.001 | 0.004 | <0.001 | >0.999 | >0.999 | 0.912 | 0.897 | >0.999 | 0.658 | 0.840 | 0.874 | 0.893 |
| FD_SR_40keV | FD_UHR_Bv48 | <0.001 | 0.481 | <0.001 | 0.157 | >0.999 | 0.827 | 0.918 | 0.377 | 0.675 | 0.002 | 0.760 | 0.672 |
| Volume-rendered | |  |  |  |  |  |  |  |  |  |  |  |  |
| FD_SR_40keV | FD_UHR_Bv48 | <0.001 | <0.001 | <0.001 | >0.999 | 0.635 | 0.258 | 0.464 | 0.040 | 0.544 | 0.486 | 0.001 | 0.005 |
| FD_SR_40keV | FD_UHR_Bv56 | <0.001 | <0.001 | <0.001 | >0.999 | 0.233 | 0.146 | 0.610 | 0.076 | 0.600 | 0.776 | 0.001 | 0.009 |
| FD_SR_40keV | FD_UHR_Bv60 | <0.001 | <0.001 | <0.001 | >0.999 | 0.366 | 0.087 | 0.380 | 0.058 | 0.389 | 0.772 | 0.021 | 0.041 |
| FD_SR_40keV | LD_UHR_Bv48 | <0.001 | <0.001 | 0.274 | >0.999 | 0.478 | 0.036 | 0.247 | <0.001 | 0.648 | 0.741 | <0.001 | 0.001 |
| FD_SR_40keV | LD_SR_40keV | 0.002 | 0.425 | 0.024 | >0.999 | 0.738 | 0.005 | 0.153 | 0.005 | 0.781 | 0.881 | 0.487 | 0.533 |
| LD_SR_40keV | FD_UHR_Bv48 | <0.001 | <0.001 | <0.001 | >0.999 | 0.490 | 0.080 | 0.480 | 0.588 | 0.751 | 0.594 | 0.012 | 0.024 |
| LD_SR_40keV | FD_UHR_Bv56 | <0.001 | <0.001 | <0.001 | >0.999 | 0.179 | 0.219 | 0.367 | 0.413 | 0.810 | 0.892 | 0.015 | 0.039 |
| LD_SR_40keV | FD_UHR_Bv60 | <0.001 | <0.001 | <0.001 | >0.999 | 0.287 | 0.320 | 0.531 | 0.500 | 0.581 | 0.890 | 0.125 | 0.136 |
| LD_SR_40keV | LD_UHR_Bv48 | <0.001 | <0.001 | 0.002 | >0.999 | 0.739 | 0.367 | 0.807 | 0.237 | 0.868 | 0.641 | 0.006 | 0.010 |
| FD_UHR_Bv48 | FD_UHR_Bv56 | <0.001 | 0.719 | <0.001 | >0.999 | 0.453 | 0.670 | 0.837 | 0.795 | 0.939 | 0.701 | >0.999 | 0.876 |
| FD_UHR_Bv48 | FD_UHR_Bv60 | <0.001 | 0.205 | <0.001 | >0.999 | 0.638 | 0.498 | 0.919 | 0.897 | 0.821 | 0.696 | 0.362 | 0.484 |
| FD_UHR_Bv48 | LD_UHR_Bv48 | 0.015 | 0.019 | 0.002 | >0.999 | 0.344 | 0.355 | 0.651 | 0.102 | 0.875 | 0.313 | 0.848 | >0.999 |
| FD_UHR_Bv56 | FD_UHR_Bv60 | 0.068 | 0.352 | 0.239 | >0.999 | 0.791 | 0.816 | 0.753 | 0.897 | 0.762 | >0.999 | 0.378 | 0.589 |
| FD_UHR_Bv56 | LD_UHR_Bv48 | <0.001 | 0.719 | <0.001 | >0.999 | 0.453 | 0.670 | 0.837 | 0.795 | 0.939 | 0.701 | >0.999 | 0.876 |
| FD_SR_40keV | FD_UHR_Bv48 | <0.001 | 0.001 | <0.001 | >0.999 | 0.207 | 0.868 | 0.714 | 0.080 | 0.696 | 0.547 | 0.265 | 0.432 |

FD = full dose, keV = kiloelectron volt, LD = low dose, SD = standard deviation, UHR = ultra-high resolution, SR = standard-reconstruction. The adjusted alpha level using Bonferroni correction is 0.05/15 = 0.003. The P values with significance were marked in red.
